# Brain Tumor Detection and Prediction in MRI Images Utilizing a Fine-Tuned Transfer Learning Model Integrated Within Deep Learning Frameworks

**DOI:** 10.3390/life15030327

**Published:** 2025-02-20

**Authors:** Deependra Rastogi, Prashant Johri, Massimo Donelli, Lalit Kumar, Shantanu Bindewari, Abhinav Raghav, Sunil Kumar Khatri

**Affiliations:** 1 School of Computer Science and Engineering, IILM University, Greater Noida 201306, India; lalitkumar@iilm.edu (L.K.); bindewarishantanu@gmail.com (S.B.); abhinav.raghav@yahoo.in (A.R.); 2 School of Computing Science and Engineering, Galgotias University, Greater Noida 203201, India; johri.prashant@gmail.com; 3 Department of Civil, Environmental, Mechanical Engineering University of Trento, 38100 Trento, Italy; 4 Radiomics Laboratory, Department of Economy and Management, University of Trento, 38100 Trento, Italy; 5 PVC Academic, Amity University, Noida 201301, India; sunilkkhatri@gmail.com

**Keywords:** brain tumor, image processing, augmentation, deep learning, transfer learning, fine-tune, InceptionResNetV2, VGG19, Xception, MobileNetV2

## Abstract

Brain tumor diagnosis is a complex task due to the intricate anatomy of the brain and the heterogeneity of tumors. While magnetic resonance imaging (MRI) is commonly used for brain imaging, accurately detecting brain tumors remains challenging. This study aims to enhance brain tumor classification via deep transfer learning architectures using fine-tuned transfer learning, an advanced approach within artificial intelligence. Deep learning methods facilitate the analysis of high-dimensional MRI data, automating the feature extraction process crucial for precise diagnoses. In this research, several transfer learning models, including InceptionResNetV2, VGG19, Xception, and MobileNetV2, were employed to improve the accuracy of tumor detection. The dataset, sourced from Kaggle, contains tumor and non-tumor images. To mitigate class imbalance, image augmentation techniques were applied. The models were pre-trained on extensive datasets and fine-tuned to recognize specific features in MRI brain images, allowing for improved classification of tumor versus non-tumor images. The experimental results show that the Xception model outperformed other architectures, achieving an accuracy of 96.11%. This result underscores its capability in high-precision brain tumor detection. The study concludes that fine-tuned deep transfer learning architectures, particularly Xception, significantly improve the accuracy and efficiency of brain tumor diagnosis. These findings demonstrate the potential of using advanced AI models to support clinical decision making, leading to more reliable diagnoses and improved patient outcomes.

## 1. Introduction

The human brain is an essential organ that coordinates perception, cognition, emotion, and behavior. Its billions of neurons form a vast network that transmits complex chemical and electrical signals. This remarkable organ has several regions that contribute to balance and coordination, including the cerebral cortex, seat of awareness, and cerebellum [1].

A tumor, scientifically known as a neoplasm, arises as an abnormal collection of cells, forming a noticeable lump or mass within the body [2]. Tumors are classified as either benign or malignant. Benign tumors typically grow slowly and remain localized without spreading to other parts of the body. However, if they press against vital organs or grow significantly in size, this can lead to complications [3]. Tumors can develop in various organs, including the brain, breast, lungs, liver, colon, and skin. In the case of brain tumors, they may originate from brain tissue or metastasize from other parts of the body [4,5]. Diagnosis often involves imaging techniques such as CT or MRI scans, along with biopsies, to determine the tumor’s nature. There are numerous types of brain tumors, including gliomas, meningiomas, pituitary tumors, schwannomas, and glioblastomas.

- Gliomas—originating from the brain’s supportive glial cells, these tumors can manifest anywhere within the brain, exhibiting varying grades of severity;
- Meningiomas—nestled within the protective layers encompassing the brain and spinal cord, these tumors typically maintain a benign nature;
- Pituitary adenomas—arising within the pituitary gland, a pivotal hormonal regulator nestled at the brain’s base, these tumors disrupt hormone production;
- Schwannomas—emerging from Schwann cells responsible for safeguarding nerve fibers with myelin sheaths, these malignancies pose a threat to nervous system integrity;
- Glioblastomas—representing the apex of glioma aggressiveness, these tumors epitomize the utmost peril within the brain tumor spectrum.

In the ever-evolving domain of neuro-oncology, the relentless pursuit of knowledge remains a cornerstone of advancements in diagnostic methodologies and therapeutic interventions, offering a ray of hope amid the complexities of cerebral pathology. The integration of cutting-edge technologies such as deep learning and artificial intelligence (AI) has revolutionized medical image analysis, driving significant progress in the identification, characterization, and management of various pathologies, including lung and breast carcinomas [6,7]. This synergy empowers healthcare professionals with enhanced decision-making capabilities, facilitating precise delineation of pathological entities and informing tailored treatment regimens to optimize patient outcomes [8,9]. At the forefront of surgical planning, artificial intelligence has become an indispensable tool, enabling meticulous segmentation of lesion boundaries and critical brain structures. By leveraging predictive analytics, AI not only anticipates potential complications but also forecasts recurrence probabilities and treatment responses, thereby guiding the formulation of personalized follow-up strategies and refining patient care pathways. Enter transfer learning (TL) [10,11], a pivotal approach in contemporary machine learning that has garnered significant attention within the medical community for its ability to leverage pre-trained models for specialized medical imaging tasks. TL streamlines the model development process by capitalizing on knowledge distilled from extensive datasets, thereby expediting model deployment while reducing computational overhead. As a key component of medical image analysis, TL utilizes renowned architectures such as VGG, ResNet, Inception, MobileNet, and DenseNet, each designed to address specific diagnostic challenges with exceptional accuracy. Delving into the technical nuances, the fusion of transfer learning with neural network architectures establishes a sophisticated framework capable of discerning intricate patterns within medical imagery with unprecedented precision. This synergy extends beyond conventional methodologies, encompassing a diverse array of specialized models poised to enhance the diagnostic capabilities of healthcare professionals. The benefits of transfer learning are evident across the patient care continuum, as early identification and accurate classification of diseased entities are crucial for the timely initiation of therapies [12]. The ongoing evolution of deep learning, artificial intelligence, and transfer learning is set to drive a revolutionary transformation in medical image analysis. This dynamic relationship has the potential to redefine the standards of patient care and diagnostic accuracy. In the present study, we embarked on a rigorous comparative analysis, evaluating the performance of four prominent transfer learning paradigms—InceptionResNetV2, VGG19, Xception, and MobileNetV2—in classifying MRI data. The cornerstone of our contribution lies in the innovative application of transfer learning methodologies, coupled with fine-tuning techniques, optimize model performance in categorizing and predicting brain neoplasms with unprecedented precision and efficiency.

- To enhance the performance of transfer learning models by refining them post-processing and then evaluating their efficacy on benchmark datasets;
- To ensure a valid comparison, by assessing the effectiveness of our refined transfer learning methodologies in contrast to prior research;
- To enable via transfer learning the utilization of pre-existing models, which is particularly advantageous in situations where there is a scarcity of labeled medical data;
- To demonstrate the efficacy of our transfer learning techniques in categorizing brain tumors, as well as their capacity to improve diagnostic precision in medical image analysis.

The research paper begins with an Introduction in Section 1 that provides context and outlines the significance of the research topic, clearly stating the problem, objectives, and scope, and briefly describing the paper’s structure. A Literature Review follows in Section 2, summarizing the existing research, discussing relevant theories and models, identifying gaps in the literature, and presenting the research hypothesis. Section 3 (Materials and Methods) details the study design, data collection methods, materials used, research procedures, and data analysis techniques. Next, Section 4 (Model Performance and Evaluation Parameters) describes the models used, defines performance metrics, presents the results of the model evaluations, and discusses validation techniques. Section 5 (Discussion and Comparison) interprets the results in the context of the research objectives, compares findings with previous studies, discusses theoretical and practical implications, acknowledges study limitations, and suggests areas for future research. Finally, the Conclusion in Section 6 summarizes key findings, highlights the study’s contributions, discusses practical applications, and provides final reflections. References and any necessary appendices are included at the end of the paper to support the research and provide additional information.

## 2. Literature Review

The categorization of brain MRI pictures has been the subject of several investigations. For example, S. Kumar et al. [13] achieved a remarkable maximum accuracy of 96.3% by feature extraction using a Deep CNN. In a different study [14], the authors achieved an remarkable 99% accuracy rate in tumor-grade categorization by recommending the use of artificial neural networks (ANNs) and other classifiers. Furthermore, extreme gradient boosting—a type of machine learning model—was applied in [15] to identify brain tumors with a remarkable 97% accuracy. Researchers used a support vector machine (SVM) classifier to perform several cross-validations on the feature set in a similar manner [16]. The suggested method’s accuracy was found to be 97.1% in a comparative study. In [17], scientists developed deep neural networks by combining a CNN with traditional architecture and a correlation learning mechanism (CLM). Their ground-breaking research shows that the CLM model attained an remarkable accuracy rate of almost 96%. In [18], scientists explored the complexities of brain tumor segmentation with the UNet model, achieving an accuracy of 89%, and they present a novel statistical strategy and machine learning method that yields an astounding accuracy rate of 98.9%. They also provide an interactive web application specifically designed to assist those who have survived brain cancer. A novel combination of deep learning and machine learning methods is presented in [19] to handle the categorization of hydrocephalus in brain CT pictures, with an impressive 98.5% accuracy rate. Achieving an astounding accuracy rate of 98.69%, the authors in [20] describe a revolutionary deep learning architecture designed especially for the classification of brain tumor pictures. Additionally, the AlexNet model is used in [21] to successfully diagnose brain tumors, demonstrating an astounding peak accuracy of 99.04%. Additionally, Ref. [22] explores the challenge of classifying tumor segments to aid in classifying brain tumors. To accomplish a 98.5% classification accuracy, the authors utilized a Jaya optimization algorithm (JOA) that is based on a deep autoencoder (DAE). This study [23] presents DIR-GAN, a deep learning method for brain tumor detection in MRI images. It uses advanced filtering, segmentation, and feature extraction to enhance classification accuracy, achieving up to 98.86% accuracy. Moreover, Ref. [24] achieved an amazing 99% accuracy rate in brain tumor picture classification, being the first to apply a deep residual network. A unique deep learning technique is offered in a pioneering initiative described in [25] for the identification and classification of microscopic brain tumors. Using a 3D CNN architecture, the strategy achieved a respectable accuracy of 92.67%. Moreover, Ref. [26] describes a novel hybrid deep technique that combines the transformer model with the self-attention unit to classify brain tumors with a previously unheard of 99.30% accuracy. The BrainMRNet model in [27] was painstakingly created to classify brain MRI images, reaching an accuracy milestone of 96.05%. Furthermore, Ref. [28] improves the VGG16 architecture to accomplish remarkable classification and detection accuracy rates of up to 98.69%. Moreover, Ref. [29] provides a novel convolutional neural network with a remarkable accuracy of 98.81%, and it is specifically designed for brain tumor MRI image segmentation. Finally, Ref. [30] introduces a novel model called Attention–Convolutional–LSTM that is designed especially for brain tumor classification, which has an amazing accuracy rate of 98.90%.

In [31], the investigators used the VGG16, InceptionV3, and ResNet50 architectures to leverage three different approaches to transfer learning. Unfortunately, among the three models, the VGG16 model came out on top with the highest accuracy of 91.58%. Using pre-trained networks like AlexNet, ResNet18, GoogleNet, and ShuffleNet, the researchers in [32] explored the depths of deep feature extraction from tumor regions and reached an amazing accuracy milestone of 98.02%. In the meantime, Ref. [33] offers a novel model with an unparalleled 99.3% accuracy rate that is specifically designed for brain tumor identification from MRI pictures. In [34], a thorough five-fold cross-validation methodology is used to evaluate the following five well-known convolutional neural networks: AlexNet, VGG16, ResNet18, GoogleNet, and ResNet50. The fluid-attenuated inversion recovery (FLAIR) MRI method performs exceptionally well, with an accuracy of 98.88%. The authors of [35] are the first to construct a CNN model that is painstakingly designed to classify different forms of brain tumors from MR images, with an impressive accuracy rate of 98.32%.

The Brain Tumor Classification-Fast Convolution Neural Network (BTC-fCNN) model is presented in Ref. [36]. Using five iterations of transfer learning, the model achieves an impressive average accuracy of 98.63%, with a remarkable 98.86% through retrained five-fold cross-validation. In [37], researchers implemented a novel endeavor by pre-training five different versions of EfficientNets, which resulted in an exceptional accuracy of 98.86% when using the suggested EfficientNet approach. EfficientNetB2 exhibited a very good performance, achieving an exceptional accuracy of 98.93% by making use of the deep convolutional neural network (DCNN) architecture VGGNet, which was pre-trained on large datasets.

The study in [38] enhanced brain tumor diagnosis using a modified ResNet50, achieving a 97.35% accuracy with strong precision and recall. Leveraging a balanced MRI dataset and data augmentation, it improves the generalizability and supports radiologists in early detection and treatment planning. Future work will focus on clinical implementation and adaptability. The authors of Ref. [39] propose an intelligent hybrid system for early brain tumor diagnosis, integrating auto contrast enhancement and deep transfer learning with Inception V3. The two-phase approach enhances MRI contrast and improves classification accuracy, achieving 98.89% on a diverse dataset. Compared to state-of-the-art models, it demonstrates superior robustness and performance, reinforcing its clinical applicability. Reference [40] utilized the YOLO NAS model for brain tumor classification, leveraging MRI images from the REMBRANDT repository. Preprocessing with HADF and segmentation via En-DeNet (U-Net + EfficientNet) enhance the accuracy. The YOLO NAS surpassed models like DNN and DenseNet-161, showcasing its potential for clinical applications.

Despite significant advancements in brain tumor classification using deep learning and machine learning models, several research gaps remain. While numerous studies have achieved high accuracy rates using CNN, ANN, SVM, and hybrid deep learning approaches, there is a lack of research focusing on optimizing pre-trained models for MRI-based tumor detection. Most studies apply models such as VGG16, ResNet, and AlexNet without extensive optimization, which could further enhance performance and generalizability. Another key gap is the lack of generalization across diverse MRI datasets. Many models demonstrate impressive accuracy on specific datasets, but their robustness in real-world clinical settings remains uncertain. Moreover, deep learning models for brain tumor classification often function as black-box systems with limited interpretability. The integration of explainable AI (XAI) techniques is necessary to enhance model transparency and clinical acceptance. While various architectures have been developed, comparative studies on different deep learning models are still insufficient, leaving uncertainties about the trade-offs between model complexity, accuracy, and computational cost. Furthermore, real-time deployment and clinical feasibility studies are underexplored, with limited discussions on hardware constraints, inference time, and real-time processing capabilities.

## 3. Materials and Methods

Four well-recognized transfer learning methodologies have been employed in this study to classify two categories, enabling the scrutiny and evaluation of our proposed framework. It leverages transfer learning architectures, including InceptionResNetV2, VGG19, Xception, and MobileNetV2. By utilizing these diverse transfer learning techniques, our dataset was rigorously examined. The dataset was partitioned into training and testing subsets based on the data distribution. This partitioning is crucial, as the training subset facilitates model learning, the validation subset aids in the model’s evaluation with sample data, and the test subset plays a key role in the comprehensive assessment of the proposed model. The presented model demonstrates confidence across multiple stages.

### 3.1. Dataset Description and Splitting

This paper used the Kaggle brain tumor dataset [41] for our analysis, which included 3762 patients’ MRI pictures—both those without brain tumors and those who had been diagnosed with them. This dataset consists of 2079 MRI pictures classified as non-tumorous (marked by 0) and 1683 images labeled as tumorous (denoted by 1).

Table 1 presents the numbers of images belonging to the two classes.

**Figure 1:**
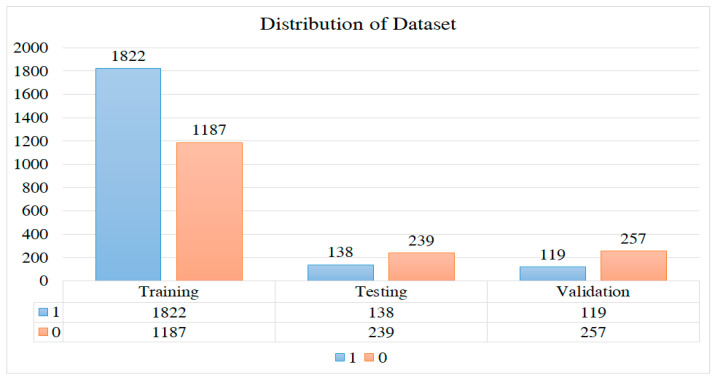
Illustrates the dataset’s distribution, showing the number of MRI images available in each class (no tumor vs. with tumor). This helps in understanding the balance of the dataset used for the model’s training.

Table 2 and Figure 1 outline the distribution of images for training, testing, and validation purposes. In total, there are 3762 images, with 3009 allocated for training, 376 for testing, and 377 for validation. These images encompass both classes 0 and 1. The ratio of images is as follows: 80% for training, 10% for validation, and 10% for testing.

**Figure 2:**
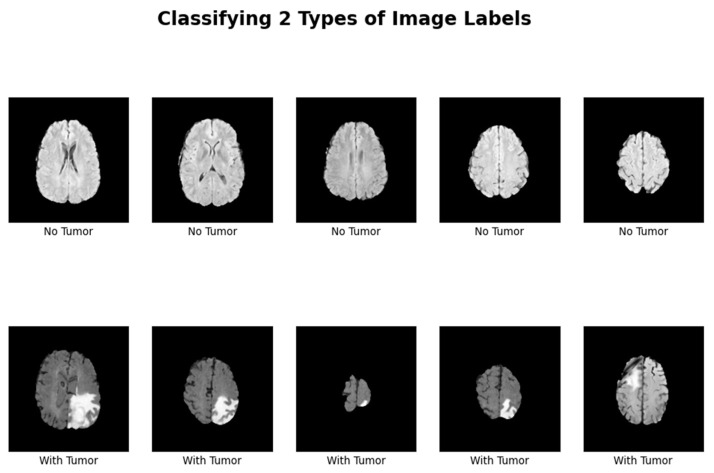
Visualization of the two representative MRI image types, with one depicting a healthy brain (no tumor) and another with a detected tumor, emphasizing the visual differences that the model needs to learn.

Figure 2 shows that images depicting no tumor typically reveal a serene landscape of brain tissue, characterized by uniform intensity and well-defined anatomical structures. MRI images exhibiting tumors offer a stark contrast. Within these scans, areas of heightened intensity or irregular masses emerge, signaling the presence of pathological growths. These tumors may manifest as distinct lesions, irregular masses, or regions of increased signal intensity, disrupting the otherwise orderly landscape of brain tissue. Notably, the presence of tumors often induces observable changes in adjacent structures, which may appear compressed, displaced, or distorted. By discerning these visual cues, healthcare professionals can effectively differentiate between images with no tumor and those with tumors, facilitating accurate diagnosis and informed treatment decisions for patients

The decision to reduce the images to 150 × 150 was made to balance computational efficiency and model performance. Deep learning models, require significant computational resources, and resizing images helps reduce memory usage and processing time while maintaining essential features for classification. Reducing the image size to 150 × 150 preserves key structural and textural information while making the training process more efficient, reducing overfitting, and allowing for faster convergence.

### 3.2. Data Augmentation

By implementing various modifications to the original images, a technique known as “image augmentation” can be used to enhance a dataset’s diversity and richness. These adjustments help preserve the semantic meaning of the images while generating new, slightly altered versions.

One common augmentation technique is rotation, where images are rotated by a certain degree clockwise or counterclockwise. This helps the model become more robust to variations in orientation that may occur in real-world scenarios. Another technique is flipping, which involves horizontally or vertically flipping images to simulate different viewpoints, enabling the model to recognize objects from multiple perspectives. Additionally, scaling can be applied to resize images to different dimensions, allowing the model to learn from images of varying sizes. Other augmentation techniques include translation, where images are shifted horizontally or vertically, and shearing, which distorts images by shifting pixels along a given axis. These modifications contribute to a more extensive training dataset, ultimately enhancing the model’s ability to generalize and perform well on previously unseen data.

**Figure 3:**
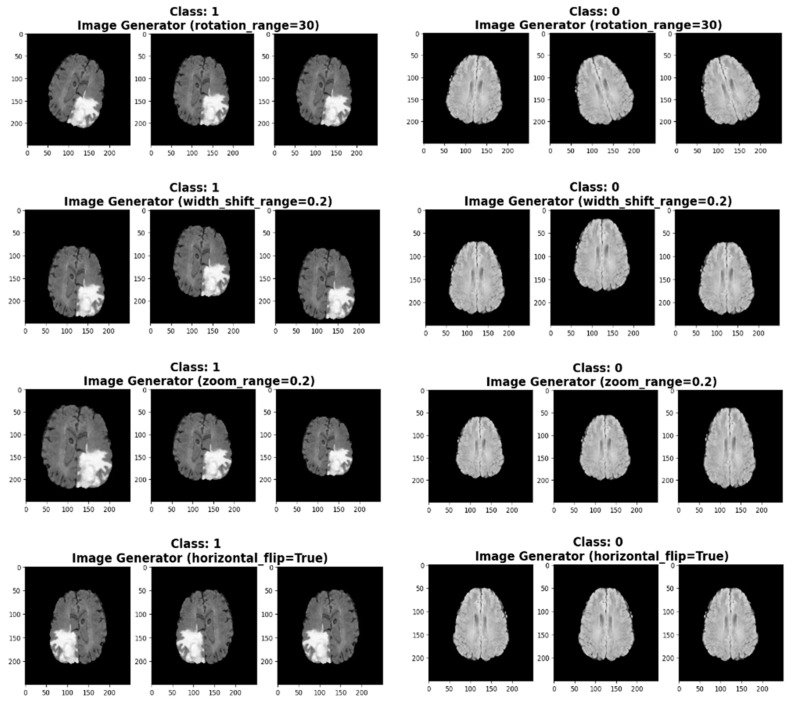
Demonstrates the augmentation techniques applied to the dataset, such as rotation, flipping, and brightness adjustment, to enhance the model’s generalizability and reduce overfitting.

Image augmentation is a crucial strategy in image processing that is aimed at reducing overfitting in machine learning models. It involves modifying the original images in various ways, thereby adding new copies to the dataset while preserving their semantic information. By exposing the model to a wider range of conditions and variations, augmentation strengthens its resilience and reduces its tendency to memorize specific elements of the training set. By increasing the diversity of the training dataset, augmentation helps the model learn invariant properties, allowing it to recognize objects in different orientations, locations, sizes, and lighting conditions. Ultimately, augmentation improves the model’s ability to generalize learned representations, leading to enhanced performance on unseen data and mitigating the risk of overfitting. Figure 3 depicts the augmentation techniques applied to the given datasets.

In machine learning workflows, the training, testing, and validation data generators are essential components for efficiently processing datasets and evaluating a model’s performance. The training data generator loads batches of training samples and applies data augmentation techniques to diversify the dataset, thereby improving the model’s ability to generalize. It also shuffles the training samples to ensure the model learns from a varied order of data in each epoch. The validation data generator, on the other hand, loads batches of validation samples without augmentation to assess the model’s performance on unseen data during training. Lastly, the testing data generator loads batches of testing samples for final evaluation, providing insight into the model’s ability to generalize to real-world data. By utilizing these data generators, practitioners can efficiently manage datasets, enhance model performance, and ensure robustness during deployment.

Algorithm 1 outlines the transformations and operations executed in the implementation and detailing the logical sequence of the steps.
**Algorithm 1** Data Augmentation and Generator Initialization
Step 1: Define Augmentation Parameter 1.Rescaling:$p^{\prime }=\frac{p}{127.5},$ for each pixel, *p*, in image. 2.Rotation: randomly select an angle $\theta \in \left[{-30}^{{}^{\circ}},\;30^{{}^{\circ}}\right],$ for each pixel position (x, y). 3.Width and Height Shifting: Randomly select a shift of $\theta \in \left[-0.2W,\;0.2W\right],\;\;t_{y}\in \left[-0.2H,\;0.2H\right],$ where W and H are the width and height of the image. Update pixel positions, as follows:$x^{\prime }=x+t_{x}$$y^{\prime }=y+t_{y}$ 4.Shear: randomly select shear angle $\theta \in \left[-0.2,\;0.2\right],$ and apply the following shear transformation:$\left[\begin{array}{c} x^{\prime }\\ y^{\prime } \end{array}\right]=\left[\begin{array}{cc} 1 & tan(\emptyset )\\ 0 & 1 \end{array}\right]\left[\begin{array}{c} x\\ y \end{array}\right]$ 5.Zoom: Randomly select a scaling factor, $z_{x}z_{y}\in \left[0.8,\;1.2\right]$. Scale the pixel positions as follows:$x^{\prime }=z_{x}.x$$y^{\prime }=z_{y}.y$ 6.Horizontal Flip: with a probability of 0.5, flip the image horizontally, as follows:$x^{\prime }=-x$$y^{\prime }=y$ 7.Fill Mode: If transformations create empty regions, fill them using the nearest pixel values.Step 2: Initialize Training, Validation, and Test GeneratorsFor each dataset (training, validation, and testing), the following steps apply: 1.Input: Directory path D, batch size B, and target size T = (150,150). 2.Transformations:    •Training: apply all augmentations, as follows:T(I)=Fill(Flip(Zoom(Shear(Rotate(Shift(Rescale(I)))))))    •Validation/Test: apply only rescaling, as follows:$I^{\prime }=\frac{I}{127.5},$ 3.Batching: Group N images from directory D into batches of size B. For each batch k, the following applies:${Batch}_{k}=\{I_{1},I_{2},I_{3},\ldots \ldots \ldots ,I_{B}\}$, $I_{i}\in D$ 4.Label Assignment: Assign binary labels, L = {0,1}, based on the directory’s class structure.Step 3: Return GeneratorsThe algorithm outputs three generators, each yielding batches of (X, Y), as follows: 1.X: transformed images of shape (B, 150,150, C), where C = 3 for RGB images. 2.Y: corresponding binary labels of shape (B,1).

### 3.3. Applied Transfer Learning Models

A model built for one task can be applied to another that is comparable but unrelated using a machine learning technique known as transfer learning. By using this technique, the weights of a pre-trained model are adjusted to suit the new task, forming the foundation of a new model. The idea behind transfer learning is that a model can use features it has learned from a large dataset to perform a new task. Transfer learning, as opposed to training a new model from scratch, saves time and resources by utilizing the information from the pre-trained model. It has achieved success in a wide range of applications, including speech recognition, image recognition, and natural language processing, enabling cutting-edge performance even with a small amount of training data. Deep learning applications, including object detection, tumor diagnosis, and image classification, have benefited greatly from transfer learning. In our investigation, four transfer learning models, each using fixed-size (150 × 150) images as input, were used. This produced a matrix structure of (150,150,3).

#### 3.3.1. InceptionResNetV2

**Figure 4:**
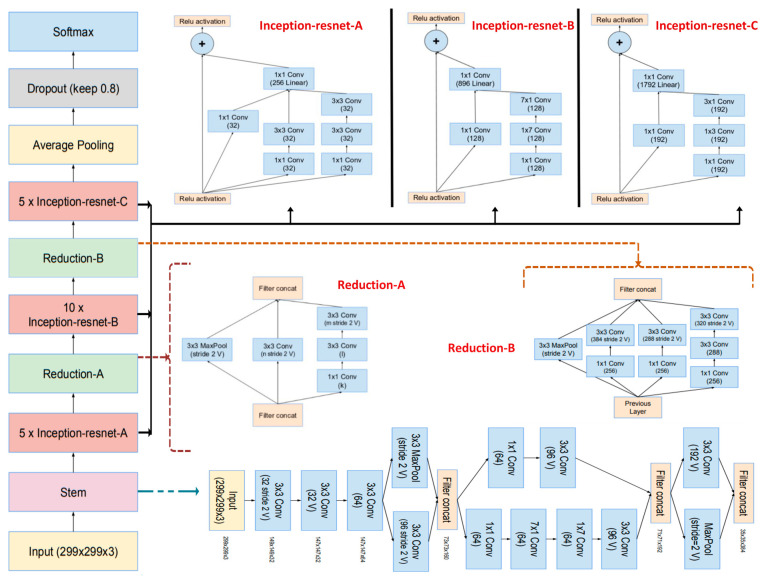
Overall architecture of the InceptionResNetV2 model, explaining its hybrid approach that combines Inception and residual connections for improved feature extraction [42].

InceptionResNetV2 (Figure 4) stands as a complex convolutional neural network (CNN) architecture primarily tailored for tasks in image classification and computer vision. It seamlessly merges the following two prominent CNN designs: Inception, recognized for its effective feature extraction, and ResNet, esteemed for its ability to tackle training challenges in deep networks. By integrating Inception modules for feature extraction and ResNet’s residual connections, Inception-ResNetV2 embodies the strengths of both approaches. Its architecture comprises stem and grid components, with the stem managing the initial image processing and the grid housing stacked Inception-ResNet modules. These modules execute multi-scale feature extraction, amalgamating diverse convolutional pathways while ensuring robust gradient flows through residual connections.

The network begins with an input image, represented as a tensor, X_input_, with dimensions of H × W × C, where *H* and *W* are the height and width of the image, and *C* is the number of channels (e.g., 3 for RGB images). This tensor is passed to the stem block, which acts as a preprocessing module that extracts low-level features while reducing the spatial dimensions.

$$X_{input}\in R^{H\times W\times C}$$

The stem block uses a series of convolutional layers, pooling layers, and activation functions to transform the input tensor. Each convolution operation applies a kernel, *W*_*i*,*c*_, to extract features for each channel, *c*, of the input, followed by the addition of a bias term, *b*_*i*_, and the application of an activation function, typically ReLU. Mathematically, the output of the convolution operation for the *i*-th feature map can be expressed as follows:

$$Z_{i}=ReLU\left(\sum_{c=1}^{C}\left(W_{i,c}*\;X_{c}\right)+b_{i}\right)$$

where ∗ represents the convolution operator. Pooling operations, such as max-pooling or average-pooling, are then applied to reduce the spatial dimensions, as follows:

$$Z_{pool}=\underset{k\times k}{\max}\left(Z\right)\;or\;{mean}_{k\times k}\left(Z\right)$$

where *k* × *k* is the pooling kernel size. The output of the stem block is a reduced spatial tensor, *Z*_stem_, that serves as the input to the Inception–Residual blocks.

The Inception–Residual blocks are the core building blocks of the InceptionResNetV2 architecture, designed to extract complex features efficiently while maintaining a smooth gradient flow through residual connections.

The Inception module processes the input, *X*, through multiple parallel branches, each performing different operations. These branches include the following:

I. 1 × 1 Convolutions to reduce dimensionality and extract fine-grained features, as follows:

$$Z_{1\times 1}=ReLU\;\left(W_{1\times 1}\times X+b_{1\times 1}\right)$$

II. 3 × 3 Convolutions with a reduction step, where a 1 × 1 convolution reduces the number of channels before applying a 3 × 3 convolution, as follows:

$$Z_{3\times 3}=ReLU\left(W_{3\times 3}\times \left(ReLU\left(W_{r}\times X+b_{r}\right)\right)+b_{3\times 3}\right)$$

III. 5 × 5 Convolutions decomposed into two sequential 3 × 3 convolutions for computational efficiency, as follows:

$$Z_{5\times 5}=ReLU\left(W_{3\times 3b}\times \left(ReLU\left(W_{3\times 3b}\times X+b_{3\times 3b}\right)\right)+b_{3\times 3b}\right)$$

IV. Pooling followed by 1 × 1 convolutions, where pooling reduces the spatial dimensions, and a 1 × 1 convolution is applied for feature compression, as follows:

$$Z_{pool}=ReLU\left(W_{pool}\times \left(Pool\left(X\right)\right)+b_{pool}\right)$$

The outputs of the parallel branches are concatenated along the channel dimension, as follows:

$$Z_{Inception}=\left[Z_{1\times 1},Z_{3\times 3},Z_{5\times 5},Z_{pool}\right]$$

The residual connection improves the gradient flow by adding the input, *X* (scaled by a weight *W*_*r*_), to the output of the Inception module, as follows:

$$Z_{Res}=Z_{Inception}+W_{r}X$$

This ensures that the network can learn to identity mappings, which helps mitigate the vanishing gradient problem.

To manage the computational complexity and memory usage, reduction blocks are placed between groups of Inception–Residual blocks. These blocks downsample the feature maps through strided convolutions and pooling operations, as follows:

I. Strided Convolutions:

$$Z_{reduce}=w_{reduce}\times X_{input}+b_{reduce}$$

II. Pooling, further reduces spatial dimensions, as follows:

$$Z_{reduce}=Pool(Z_{reduce})$$

The reduction block outputs a feature map, *Z*_reduce_, with smaller spatial dimensions but a larger number of channels.

The output of the final Inception–Residual block is flattened into a 1D vector, as follows:

$$Z_{flatten}=Flatten(Z_{final})$$

where $Z_{final}\in R^{H_{f}\times W_{f}\times C_{f}}$ and the flattened vector $Z_{flatten}\in R^{H_{f}.W_{f}.C_{f}}$

This vector is passed through one or more fully connected layers, as follows:

$$Z_{dense}=W_{dense}\;.\;Z_{final}+b_{dense}$$

where *W*dense and *b*dense are the weights and biases of the dense layer.

The final layer applies a softmax activation function to map the logits to probabilities, as follows:

$$P\left(y_{i}\right|X)=\frac{exp\left(Z_{i}\right)}{\sum_{j=1}^{N}exp\left(Z_{j}\right)}$$

where *Z*_*i*_ is the logit for class *i*, *N* is the number of output classes, and *P*(*y*_*i*_∣*X*) is the predicted probability for class *i*.

#### 3.3.2. VGG19

**Figure 5:**
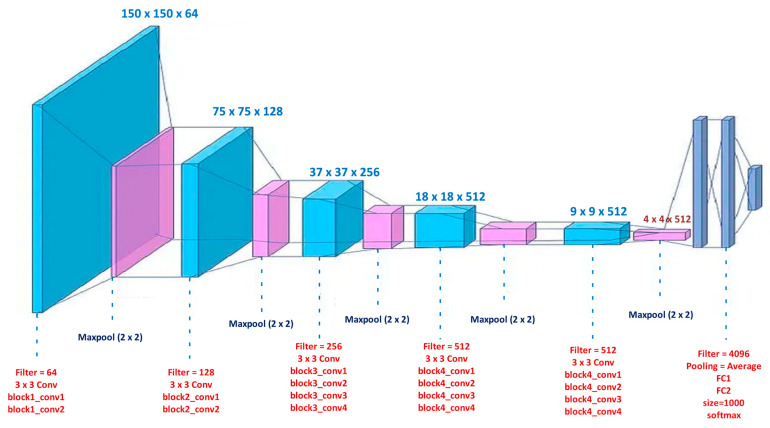
Architecture for VGG19, highlighting its deep structure with sequential convolutional layers, which contribute to hierarchical feature learning [43].

Renowned for its simplicity and depth, the Visual Geometry Group at the University of Oxford created the convolutional neural network architecture known as VGG19 [Figure 5]. VGG19, which consists of a total of 19 layers—16 convolutional layers and three fully connected layers—has become well-known for its performance in demanding image recognition applications. The convolutional layers of the design use 1-pixel stride 3 × 3 filters, which are padded to preserve spatial dimensions. Max-pooling layers with a 2 × 2 window and a stride of 2 pixels are inserted between the convolutional layers to enable spatial downsampling while maintaining important features. Beyond the convolutional layers, VGG19 features fully connected layers with 4096 neurons each, enabling the network to learn high-level representations from the extracted features. The output layer, equipped with a softmax activation function, produces class probabilities for the 1000 classes in the ImageNet dataset. Despite its simplicity, VGG19 achieved exceptional performance on image classification tasks, setting benchmarks in the field. However, its extensive parameter count renders it computationally demanding, limiting its deployment in resource-constrained environments. Nonetheless, VGG19 remains a foundational model in the evolution of convolutional neural networks, inspiring subsequent architectures with its clear and scalable design principles.

In the VGG19, first, the images *A*, *B*, and *C* are preprocessed to ensure they are in the correct format (resize, normalize, etc.).

Let *I_A_*, *I_B_*, and *I_C_* be the raw input images with dimensions *H* × *W* × 3 (height, width, and 3 color channels).

Each image is preprocessed, as follows:

$$I_{A}^{\prime }=resize\left(I_{A}\right)$$

$$I_{B}^{\prime }=resize\left(I_{B}\right)$$

$$I_{C}^{\prime }=resize\left(I_{C}\right)$$

Each image is normalized, as follows:

$$I_{A}^{\prime }=I_{A}^{\prime }-\mu$$

$$I_{B}^{\prime }=I_{B}^{\prime }-\mu$$

$$I_{C}^{\prime }=I_{C}^{\prime }-\mu$$

where *μ* is the mean vector of the image dataset.

- **Layer 1: Conv1-1**

Convolution of image $I_{A}^{\prime }$ with filter $W_{1}^{1}$ and bias $b_{1}^{1}$, as follows:

$$F_{A}^{1}=\sigma \left(W_{1}^{1}*I_{A}^{\prime }+b_{1}^{1}\right)$$

For B and C, apply as follows:

$$F_{B}^{1}=\sigma \left(W_{1}^{1}*I_{B}^{\prime }+b_{1}^{1}\right)$$

$$F_{C}^{1}=\sigma \left(W_{1}^{1}*I_{C}^{\prime }+b_{1}^{1}\right)$$

where ∗ represents the convolution operation, and *σ* is the ReLU activation function.

- **Layer 2: Conv1-2**

Convolution of image $I_{A}^{\prime }$ with filter $W_{2}^{1}$ and bias $b_{2}^{1}$, as follows:

$$F_{A}^{1}=\sigma \left(W_{2}^{1}*I_{A}^{\prime }+b_{2}^{1}\right)$$

For B and C, the following equations are applies:

$$F_{B}^{2}=\sigma \left(W_{2}^{1}*I_{B}^{\prime }+b_{2}^{1}\right)$$

$$F_{C}^{2}=\sigma \left(W_{2}^{1}*I_{C}^{\prime }+b_{2}^{1}\right)$$

- **Max Pooling (after Conv1-2)**

Apply max-pooling, as follows:

$$F_{A}^{1_{pooled}}=maxpool\left(F_{A}^{2}\right)$$

$$F_{B}^{1_{pooled}}=maxpool\left(F_{B}^{2}\right)$$

$$F_{C}^{1_{pooled}}=maxpool\left(F_{C}^{2}\right)$$

Each subsequent block of the VGG19 architecture will apply a similar set of convolutional layers followed by ReLU activations and max-pooling. After the final convolutional block, flatten the pooled feature maps to 1D vectors.

$$F_{A}^{flattened}=flatten\left(F_{A}^{L_{pooled}}\right)$$

$$F_{B}^{flattened}=flatten\left(F_{B}^{L_{pooled}}\right)$$

$$F_{C}^{flattened}=flatten\left(F_{C}^{L_{pooled}}\right)$$

where $L_{pooled}$ refers to the last pooling layer’s output. Pass the flattened feature maps through fully connected layers.

For each fully connected layer, *i*, apply the following:

$$F_{A}^{i}=W^{i}F_{A}^{flattened}+b^{i}$$

$$F_{B}^{i}=W^{i}F_{B}^{flattened}+b^{i}$$

$$F_{C}^{i}=W^{i}F_{C}^{flattened}+b^{i}$$

For the aforementioned equation, the following definitions are made

- $W^{i}$ is the weight matrix for the i-th fully connected layer;
- $b^{i}$ is the bias vector for the i-th fully connected layer.

After passing through the fully connected layers, apply the softmax function to obtain the class probabilities. For each image *A*, *B*, and *C*, the softmax function is applied to the final output, *F*^*f*^, from the last fully connected layer, as follows:

$$P_{A}=softmax\left(F_{A}^{f}\right)$$

$$P_{B}=softmax\left(F_{B}^{f}\right)$$

$$P_{C}=softmax\left(F_{C}^{f}\right)$$

The softmax function is defined as follows:

$$P_{c}=\frac{e^{F_{c}}}{\sum_{k=1}^{K}e^{F_{k}}}$$

For the aforementioned equation, the following definitions are made:

- $P_{c}$ is the probability of class *C*;
- $F_{c}$ is the score for class *C*;
- *K* is the number of classes.

#### 3.3.3. Xception

**Figure 6:**
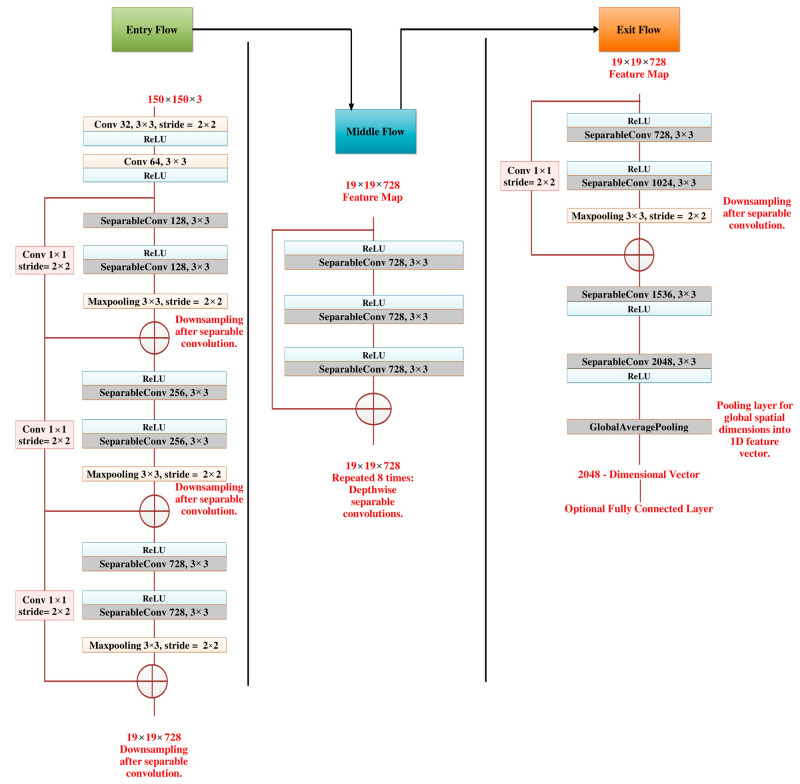
Flow of the Xception architecture, illustrating the depthwise separable convolutions that optimize computational efficiency while maintaining high accuracy [44].

Xception (Figure 6), an advanced neural network architecture developed by researchers at Google, stands out for its innovative incorporation of depthwise separable convolutions. Departing from traditional convolutional layers, Xception introduces a novel concept where Inception modules serve as a transitional step, connecting standard convolution with depthwise separable convolution operations. This perspective views depthwise separable convolutions as Inception modules expanded with numerous towers, leading to the proposal of a new convolutional neural network design inspired by the Inception model. In Xception, data flows through the entry, middle, and exit flows, with the middle flow iterated eight times. Notably, batch normalization is applied after each Convolution and SeparableConvolution layer, ensuring stable and efficient training throughout the network.

Depthwise separable convolution, a pivotal component in modern neural network architectures, involves two distinct operations: depthwise convolution and pointwise convolution. In depthwise convolution, individual filters are applied independently to each channel of the input feature map, capturing spatial information within each channel. This step effectively reduces computational complexity by processing spatial features independently across channels. Following depthwise convolution, pointwise convolution is employed to mix and transform the output channels using 1 × 1 convolutional filters. Unlike traditional convolutional layers, where filters span across all input channels, pointwise convolution allows the network to learn linear combinations of the features extracted by the depthwise convolution. By separating spatial and cross-channel information, depthwise and pointwise separable convolutions significantly reduce the computational cost of standard convolutions while preserving representational capacity. This architectural innovation [Algorithm 2] has proven instrumental in developing efficient and scalable neural network models, particularly in resource-constrained environments such as mobile and embedded devices, for which computational efficiency is paramount.
**Algorithm 2** Steps for Xception Neural Network ArchitectureStep-1 Initial Convolution Layer$Y_{1}=\sigma \left(BN\left(Conv2D\left(X,\;W_{1},b_{1},s=2\right)\right)\right)$Step-2 Each Depthwise Separable Convolution Follows:$Y=\sigma \left(BN\left(DepthwiseConv2D\left(X,\;W_{d},s\right)\right)\right)$$Y=\sigma \left(BN\left(PointwiseConv2D\left(Y,\;W_{p}\right)\right)\right)$$Y_{res}=Conv2D\left(X,\;W_{res},b_{res},s=2\right)$$Y=Y+Y_{res}$Step-3 For i = 1 to 8:$Y=\sigma \left(BN\left(DepthwiseConv2D\left(X,\;W_{d}\right)\right)\right)$$Y=\sigma \left(BN\left(PointwiseConv2D\left(Y,\;W_{p}\right)\right)\right)$Step-4 Exit Flow$Y=\sigma \left(BN\left(DepthwiseConv2D\left(X,\;W_{d},s=2\right)\right)\right)$$Y=\sigma \left(BN\left(PointwiseConv2D\left(Y,\;W_{p}\right)\right)\right)$$Y=\sigma \left(BN\left(DepthwiseConv2D\left(X,\;W_{d},s=1\right)\right)\right)$$Y=\sigma \left(BN\left(PointwiseConv2D\left(Y,\;W_{p}\right)\right)\right)$Step-5 Global Average Pooling$Y_{GAP}=\frac{1}{N}\sum_{i=1}^{N}Y_{i}$Step-6 Fully Connected Layer$Y_{output}=Softmax\left(W_{f}\cdot Y_{GAP}+b_{f}\right)$Note the following: •  X is the input (150 × 150 × 3); •  $W_{1}$ are convolution filters; •  $b_{1}$ is the bias; •  BN is batch normalization; •  ReLU is the activation function; •  $W_{d}$ are depthwise convolution filters; •  $W_{p}$ are pointwise convolution filters; •  $W_{f}$ is the weight matrix; •  $b_{f}$ is the bias vector.

#### 3.3.4. MobileNetV2

**Figure 7:**
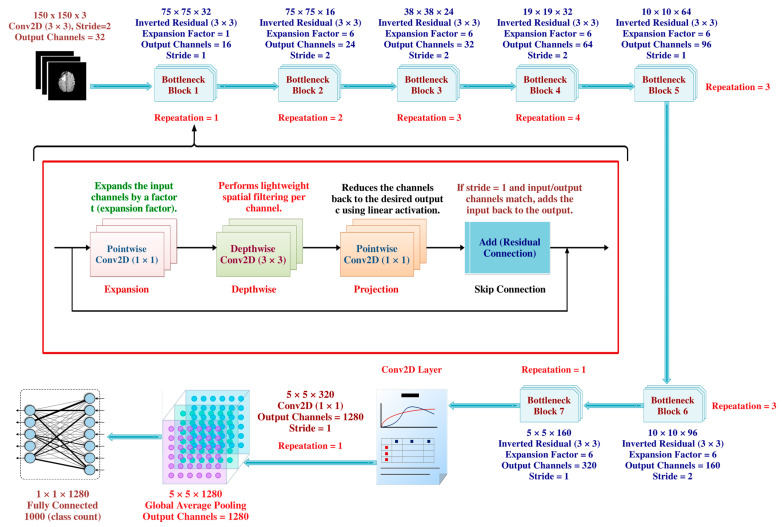
MobileNetV2 Architecture, emphasizing its use of inverted residual blocks and depthwise separable convolutions to achieve a balance between efficiency and accuracy [45].

A notable development in convolutional neural network topologies for mobile and embedded devices with constrained computational resources is represented by MobileNetV2 (Figure 7). Released in 2018, MobileNetV2 is a project by Google researchers that expands on the framework of MobileNetV1 while incorporating novel design ideas to boost efficiency and performance. At its core, MobileNetV2 leverages inverted residuals and linear bottlenecks to achieve these goals. The concept of inverted residuals involves employing bottleneck layers with low-dimensional representations between expanded layers, reducing computational complexity while maintaining representational capacity. Additionally, linear bottlenecks further enhance efficiency by reducing the number of channels in feature maps before subsequent convolutional operations. MobileNetV2 also incorporates depthwise separable convolutions and global average pooling to streamline computation and produce final predictions. With its balance of efficiency and accuracy, MobileNetV2 has become a popular choice for a wide range of computer vision tasks on mobile devices, IoT devices, and embedded systems.

#### 3.3.5. Adapted Methodology

The methodology begins by acquiring the brain tumor dataset from Kaggle, a well-known platform for sharing data in various domains. Once the dataset is acquired, it is split into the following three parts: training, validation, and testing sets. This split is crucial in deep learning to ensure that the model can generalize well to unseen data. Mathematically, the dataset, N, is divided such that 80% of the data are allocated for training, 10% for validation, and 10% for testing. This can be represented as follows:

$$N_{train}=0.80\times N$$

$$N_{val}=0.10\times N$$

$$N_{test}=0.10\times N$$

Here, the training set is utilized to train the model, the validation set is employed for tuning hyperparameters and assessing intermediate performance, and the test set is reserved for the final evaluation, ensuring unbiased model performance assessment.

To enhance the diversity of the training data and address the issue of overfitting—a scenario in which the model performs well on training data but poorly on unseen data—image augmentation is applied using an Image Data Generator. Image augmentation introduces randomness into the training process by transforming the images through operations like rotations, zooms, and flips. These transformations expand the dataset’s artificially without adding new images, making the model more robust. The images are resized to 150 × 150, 150 × 150, 150 × 150 pixels to match the input size expected by pre-trained convolutional neural networks (CNNs). During training, batches of 32 images are fed into the network, and since the problem is binary (tumor vs. non-tumor), the class mode is set to ‘binary’.

Next, several state-of-the-art pre-trained CNN architectures are loaded. These architectures include InceptionResNetV2, VGG19, Xception, and MobileNetV2, which are renowned for their high performance in computer vision tasks. These models have already been trained on massive datasets like ImageNet, so their initial layers can effectively capture general features from the images. Transfer learning is applied here, where the knowledge gained by these models from prior tasks (e.g., recognizing thousands of objects) is transferred to the new task of classifying brain tumors. The mathematical formulation of transfer learning can be expressed as follows:

$$y=f(W_{base}+W_{new}(X))$$

where $W_{base}$ are the weights from the pre-trained network that capture generic image features, $W_{new}$ are the newly learned weights tailored to the brain tumor classification task, and X represents the input MRI image.

Fine-tuning is a crucial phase in transfer learning, where specific layers of a pre-trained deep learning model are selectively updated to adapt the model to a new domain, such as MRI brain tumor classification. This step is essential because pre-trained models, such as InceptionResNetV2, VGG19, Xception, and MobileNetV2, are originally trained on large-scale datasets like ImageNet, which contain general image features such as edges, textures, and object shapes. However, MRI brain images have domain-specific patterns, such as tumor textures, contrasts, and intensities, which are not entirely represented in the original pre-trained dataset. Fine-tuning ensures that the model retains the useful generic features learned from ImageNet while adapting to the unique characteristics of MRI scans

During fine-tuning, instead of freezing all pre-trained layers, layers from the 100th onward are “unfrozen”, allowing their weights to be updated during training. This can be expressed as follows:

$$W_{trainable}=W_{100}$$

where $W_{100}$ represents the trainable parameters from the 100th layer onward. The earlier layers (below the 100th) remain frozen because they contain low-level features such as edges and textures, which are transferable across different datasets.

The network is then trained using the Adam optimizer, which is a variant of stochastic gradient descent that adapts the learning rate based on the first and second moments of the gradients. The learning rate is initially set to α = 0.0001, which controls how much the model’s weights are updated during training. The loss function employed is binary cross-entropy, which is suitable for binary classification problems. This loss function is defined as follows:

$$L=-\frac{1}{N}\sum_{i=1}^{N}\left[y_{i}log\left({\hat{y}}_{i}\right)+\left(1-y_{i}\right)log\left(1-{\hat{y}}_{i}\right)\right]$$

Here, $y_{i}$ represents the true label for each image (1 for tumor, 0 for non-tumor), and ${\hat{y}}_{i}$ is the model’s predicted probability that the image contains a tumor. The loss function penalizes incorrect predictions, with larger penalties for confident yet incorrect predictions. Once fine-tuning is complete, the model is retrained using the same binary cross-entropy loss function and Adam optimizer. The final evaluation of the model is performed using the test set, which has been kept separate from the training and validation processes to ensure an unbiased assessment of the model’s true performance. The accuracy and other performance metrics, such as precision and recall, are compared across the four architectures (InceptionResNetV2, VGG19, Xception, and MobileNetV2) to determine which model performs best in classifying brain tumors. This approach demonstrates how transfer learning, combined with fine-tuning and robust optimization techniques, can significantly enhance the accuracy of brain tumor diagnosis.

Fine-tuning allows the pre-trained model to adapt to domain-specific patterns in MRI images while preserving useful features from ImageNet. By unfreezing only the top layers, using a reduced learning rate, and applying regularization, the model is trained efficiently without overfitting, ensuring optimal performance for brain tumor classification. Table 3 is represent the hyperparameter of all the transfer learning models.

**Figure 8:**
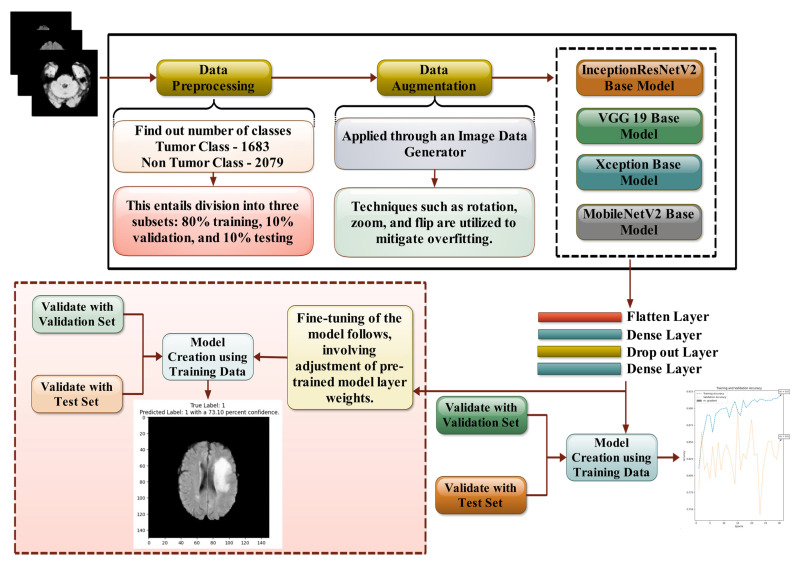
Depicts the proposed model workflow, illustrating the end-to-end process from data preprocessing to classification, demonstrating how the transfer learning models were implemented.

Figure 8 shows that the following layers are added:

- Flatten Layer: Flattening is the process of reducing data to a one-dimensional collection for further processing. In this work, a single lengthy feature representation is produced by flattening the output of the convolutional layers. Additionally, it is linked to the last classification model, commonly referred to as a fully connected layer.
- Dense Layer: A “dense layer” is a basic layer of neurons where each neuron is connected to all neurons in the layer before it, providing information to all of them. Utilizing the outcome of convolution layers, features are identified using dense layers.
- Drop out Layer: A method of eliminating neurons from a neural network or neglecting them during development is called dropout. Stated differently, individual neurons have just been removed from the network.

## 4. Model Performance and Evaluation Parameter

Adam Optimizers: The Adam optimizer, an enhanced form of stochastic gradient descent, might be used in a variety of computer vision algorithms such as computer vision applications processing. The Adam optimizer in deep learning involves several mathematical operations to update the parameters of a neural network during training. At each iteration t, the algorithm calculates the gradient of the loss function J(θ) with respect to the parameters θ. These gradients are denoted as $g_{t}=\nabla_{\theta }\;J\;\left(\theta_{t-1}\right)$, where $\theta_{t-1}$ represents the parameters at the previous iteration. Then, Adam maintains two moment vectors, $m_{t}$ and $v_{t}$, which are exponentially decaying averages of the gradients and the squared gradients, respectively. These are updated using the following equations:

$$m_{t}=\beta_{1}m_{t-1}+\left(1-\beta_{1}\right)g_{t}$$

$$v_{t}=\beta_{2}v_{t-1}+\left(1-\beta_{2}\right)g_{t}^{2}$$

where $\beta_{1}$ and $\beta_{2}$ are the exponential decay rates for the moment estimates.

To address bias in the moment estimates, Adam calculates bias-corrected moment estimates, as follows:

$${\hat{m}}_{t}=\frac{m_{t}}{1+\beta_{1}^{t}}$$

$${\hat{v}}_{t}=\frac{v_{t}}{1+\beta_{2}^{t}}$$

Finally, the parameters, θ, are updated using the bias-corrected moment estimates and the learning rate, α, as follows:

$$\theta_{t}=\theta_{t-1}-\frac{\alpha }{\sqrt{{\hat{v}}_{t}}+\epsilon }{\hat{m}}_{t}$$

where *ϵ* is a small constant added for numerical stability.

Cross-Entropy Loss Function: The Cross-Entropy Loss Function measures the discrepancy between the actual likelihood distribution among the classes and the anticipated probability distribution, and it is often utilized in artificial intelligence and deep learning classification tasks. It is particularly well-liked in scenarios in which the results are shown as the probability for several classifications. Cross-entropy may be determined in classification model when the number of categories, M, equals two, as follows:

$$-\left(ylog\right.\left(p\right)+\left(1-y\right)\log(1-p))$$

If M > 2 (multiclass categorization), estimate a loss with each target class per observation separately and add the results.

$$-\sum_{c=1}^{M}y_{o,c}\;\log(P_{o,c})$$

Accuracy: How successfully a model uses the input or training data to identify patterns and correlations among parameters in a dataset is what determines the model’s accuracy.

$$ACC=\frac{TP+TN}{TP+TN+FP+FN}$$

Based on these metrics, we can make the following observations:

○ Xception has the highest training accuracy (0.9611), indicating that it learns the training data well. It also has the lowest training loss (0.0925), suggesting that it minimizes errors during training;
○ VGG19 has the second-highest training accuracy (0.9246) and lowest training loss (0.1852). However, its validation accuracy (0.8511) and validation loss (0.3495) are comparatively lower and higher, respectively, suggesting that it may not generalize as well to unseen data;
○ InceptionResNetV2 and MobileNetV2 have similar validation accuracies and loss values, with InceptionResNetV2 having a slightly higher accuracy but also a slightly higher loss.

The strengths of a Fine-Tuned Transfer Learning Xception Deep Learning model include the following:

- High Performance: Xception is a state-of-the-art deep convolutional neural network (CNN) that has demonstrated exceptional performance in various computer vision applications, including object detection and image classification. Its advanced architecture, which replaces traditional convolutional layers with depthwise separable convolutions, significantly enhances computational efficiency without compromising accuracy. When fine-tuned for a specific task in mathematics education, Xception’s strong feature extraction capabilities allow it to identify patterns in mathematical symbols, equations, and diagrams with high precision, making it a powerful tool for educational AI applications.
- Effective Feature Extraction: One of Xception’s key strengths is its ability to capture hierarchical features from input images efficiently. The model processes data through depthwise separable convolution layers, which independently analyze spatial and depthwise information. This design enhances its ability to learn rich representations of complex patterns within mathematical content, including handwritten equations, geometric shapes, and structured graphs. As a result, Xception can accurately distinguish between similar-looking symbols and notations, improving recognition tasks in mathematics-related applications.
- Transfer Learning Advantage: By leveraging a pre-trained Xception model on large-scale datasets such as ImageNet, valuable knowledge from extensive image classification tasks can be transferred to the domain of mathematics education. Fine-tuning the model allows it to adapt to specific mathematical datasets, reducing the need for extensive labeled data. This transfer learning approach accelerates training convergence, improves performance, and enhances the model’s ability to generalize to new mathematical problems with minimal computational overhead.
- Scalability: Xception’s architecture is inherently scalable, meaning it can process images of various sizes and resolutions without requiring significant modifications. This flexibility is particularly beneficial for mathematical applications, where input data may range from simple equations and arithmetic problems to complex graphs, geometric diagrams, and multi-step problem visualizations. The ability to handle diverse mathematical content without extensive preprocessing makes Xception a highly adaptable model for educational AI solutions.
- Robustness to Variations: Mathematical expressions, diagrams, and visual content often appear in different layouts, orientations, and formats. Xception’s depthwise separable convolutions allow it to capture spatial correlations across different regions of an image, making it highly robust to variations in scale, rotation, and occlusion. This robustness ensures consistent performance even when mathematical content appears in handwritten notes, scanned textbooks, or digital whiteboards, making it a reliable model for real-world educational applications.

While fine-tuned transfer learning models, such as those based on Xception architecture, offer significant advantages, they may also present certain weaknesses or challenges in future work, as follows:

- Overfitting: Overfitting can occur when a model is fine-tuned using a particular dataset, particularly if the dataset is limited or not representative of the target population. As a result, the model may perform well on training data but badly on fresh, untested data. Regularization strategies, data augmentation, or gathering more varied training data may be necessary to address overfitting.
- Domain Shift: The source domain, where the pre-trained model was trained, and the target domain in mathematics education may be very different. The fine-tuned model may perform worse as a result of this domain shift since it may find it difficult to adjust to the subtleties and variability seen in educational data. Techniques for do-main adaption or gathering domain-specific data for optimization may be used to mitigate domain shift.
- Limited Generalization: Transfer learning may restrict the model’s capacity to generalize to completely new problems or domains within mathematics education, even while it can speed up learning by using information from the source domain. Optimized models may perform well on tasks that are similar to the ones they were trained on, but they might not perform well on tasks that call for new ideas or methods of problem solving.
- Model Interpretability: It can be difficult to understand the decision-making processes of deep learning models, especially refined transfer learning models, as they are frequently viewed as “black boxes”. The absence of model interpretability may be a major obstacle to adoption and comprehension in educational settings, where openness and interpretability are essential for confidence and acceptance.
- Data Bias and Fairness: Large-scale datasets used to train pre-trained models may contain biases that the models are unable to detect, which can result in unjust outcomes or biased predictions, particularly for underrepresented groups. The process of fine-tuning such models using biased data has the potential to increase achievement gaps and disparities by perpetuating or exacerbating pre-existing biases in mathematics education.
- Computational Resources: Large-scale datasets and high-performance GPUs or TPUs are necessary for fine-tuning deep learning models, especially those with complicated architectures like Xception. In educational contexts, access to these materials can be restricted, which would make it more difficult to create and implement transfer learning models that are optimized.

## 5. Discussion and Comparison

**Figure 9:**
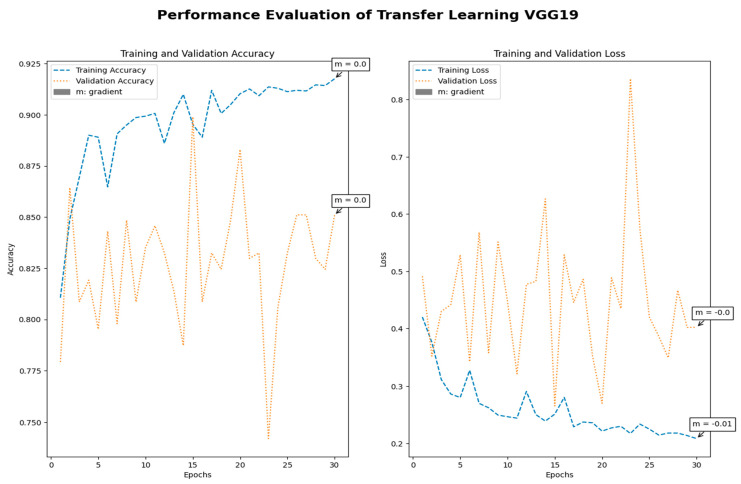
Performance assessment of transfer learning: VGG19.

**Figure 10:**
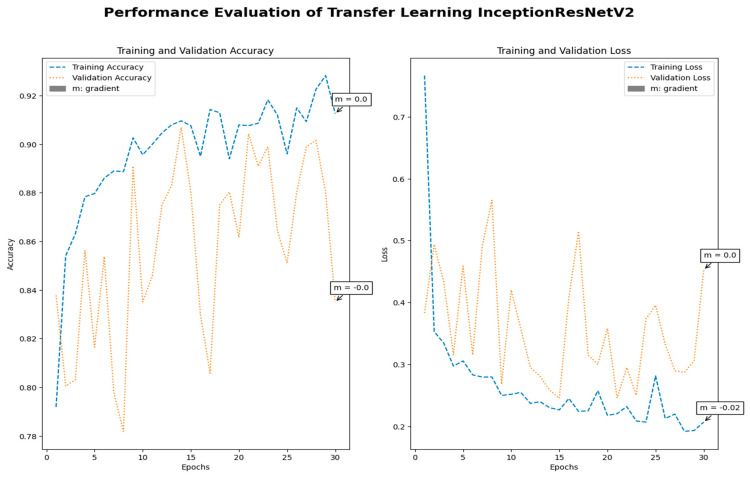
Performance assessment of transfer learning: InceptionResNetV2.

**Figure 11:**
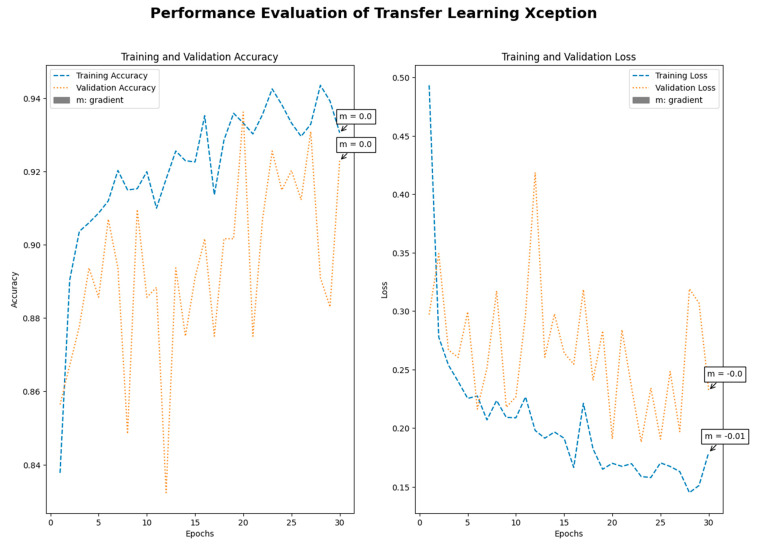
Performance assessment of transfer learning: Xception.

**Figure 12:**
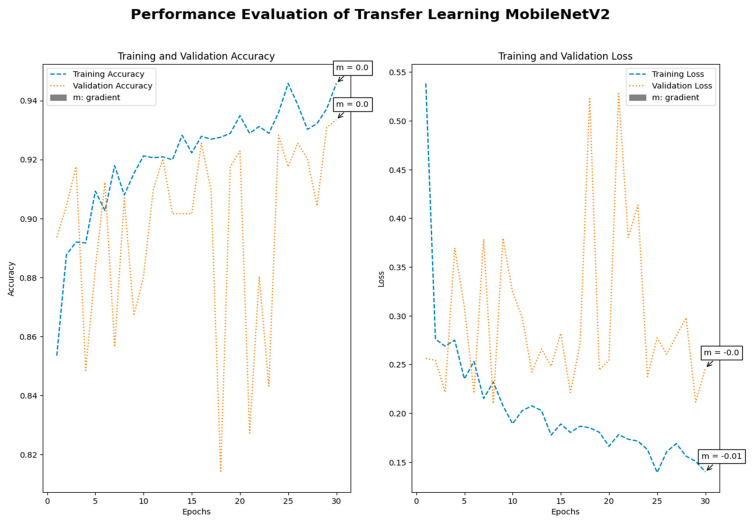
Performance assessment of transfer learning: MobileNetV2.

**Figure 13:**
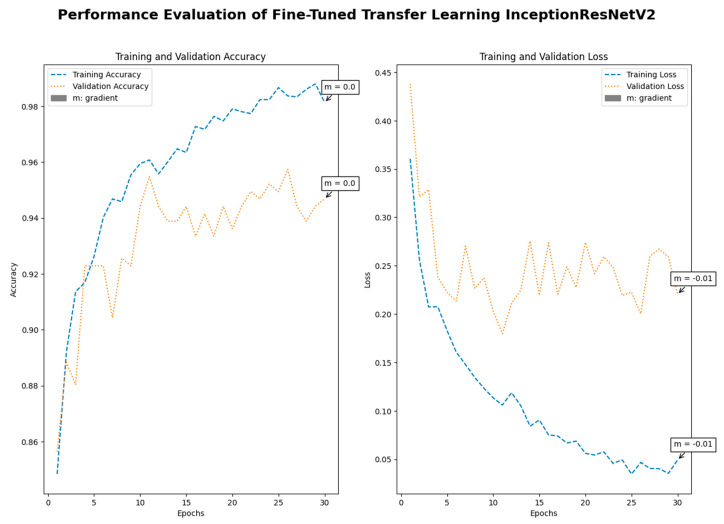
Performance assessment of fine-tuned transfer learning: InceptionResNetV2.

**Figure 14:**
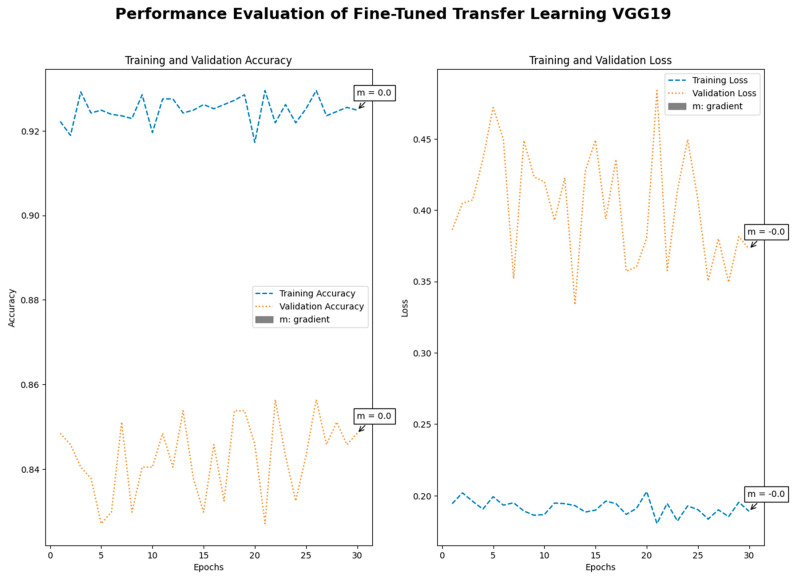
Performance assessment of fine-tuned transfer learning: VGG19.

**Figure 15:**
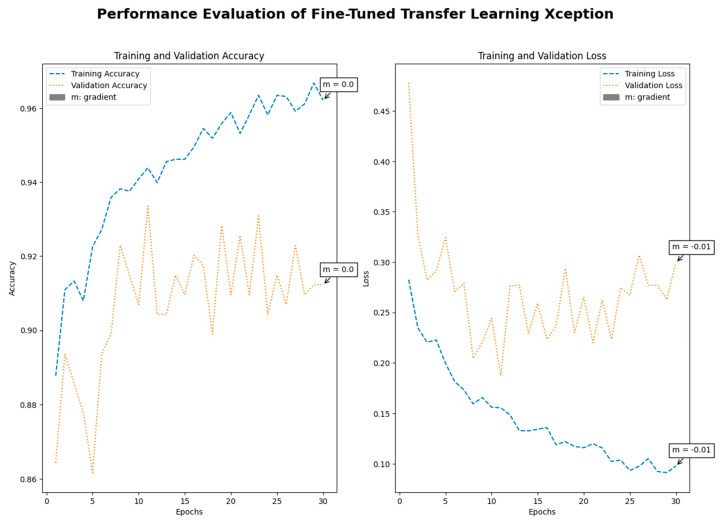
Performance assessment of fine-tuned transfer learning: Xception.

**Figure 16:**
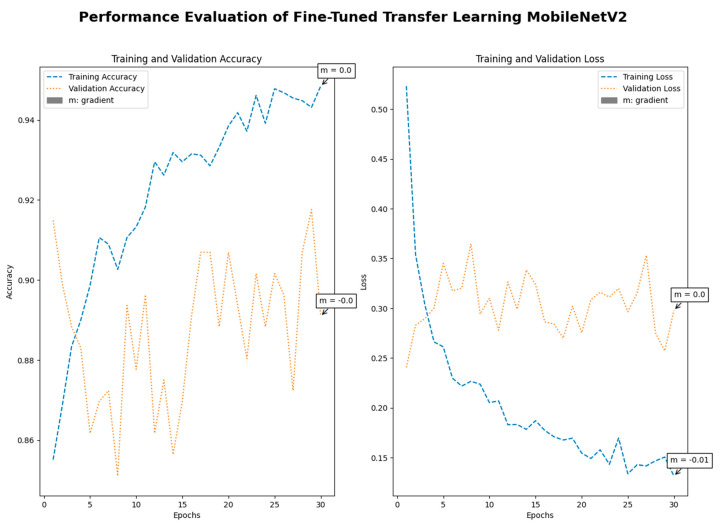
Performance assessment of fine-tuned transfer learning: MobileNetV2.

**Figure 17:**
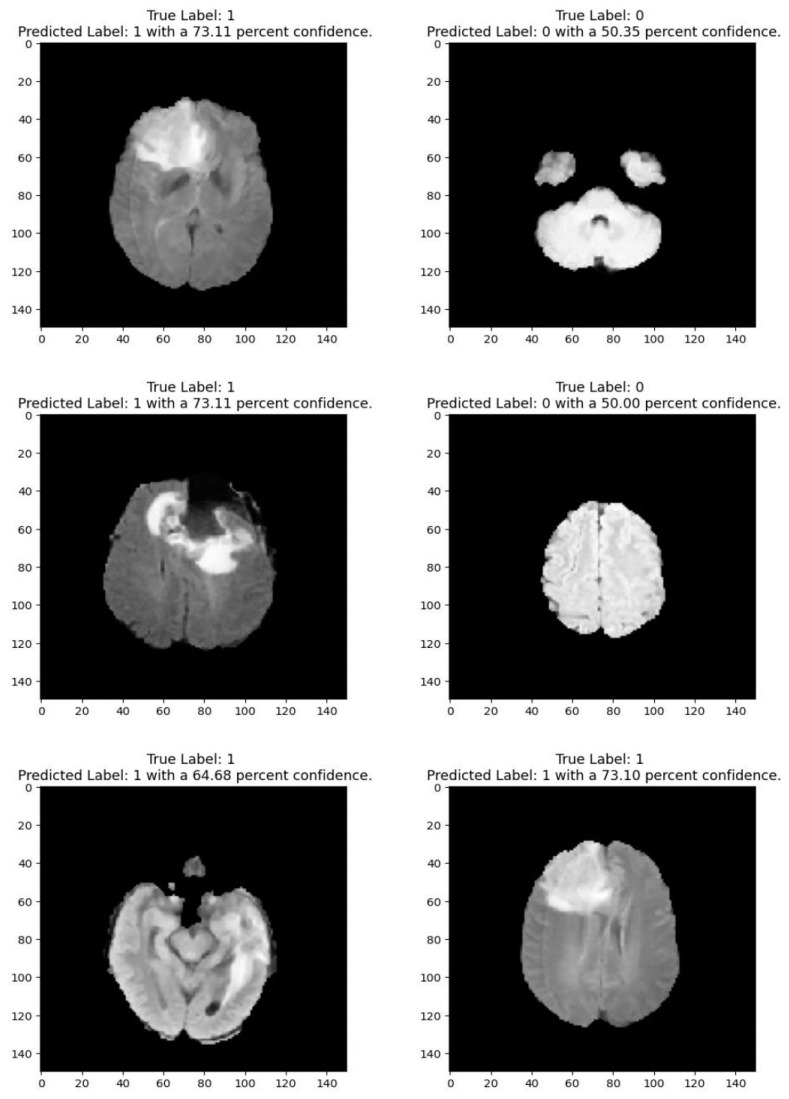
Prediction for tumor (1/0) using fine-tuned transfer learning: MobileNetV2.

**Figure 18:**
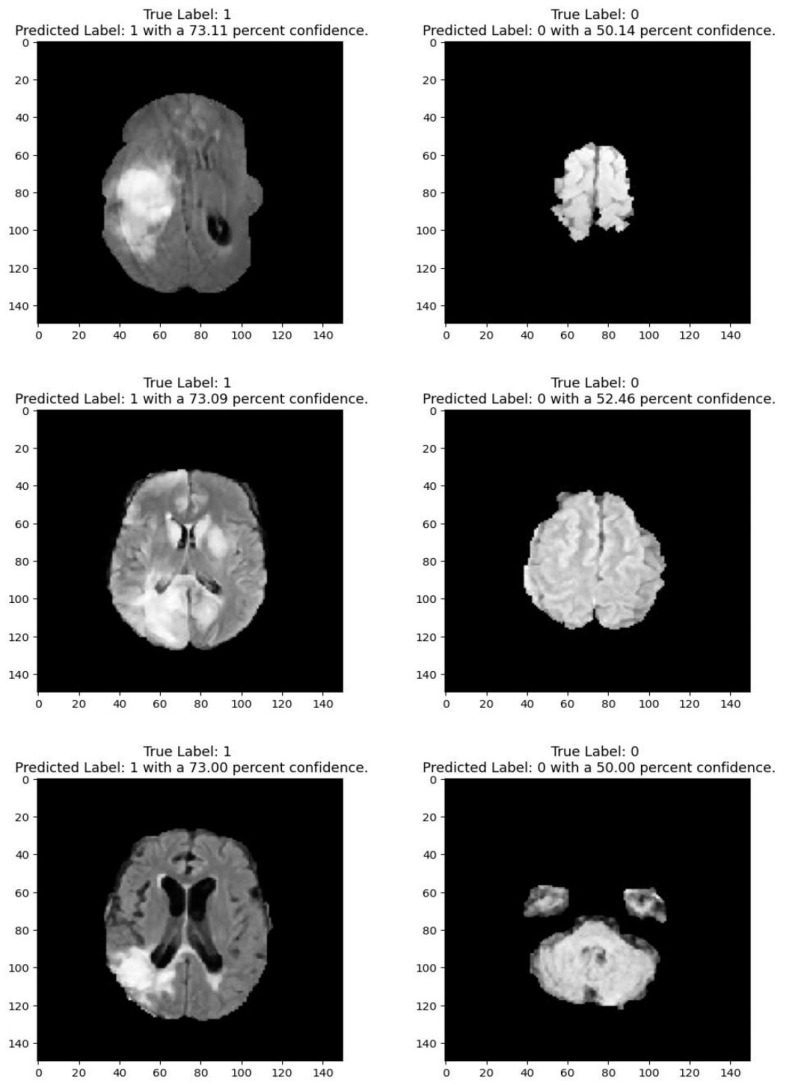
Prediction for tumor (1/0) using fine-tuned transfer learning: Xception.

**Figure 19:**
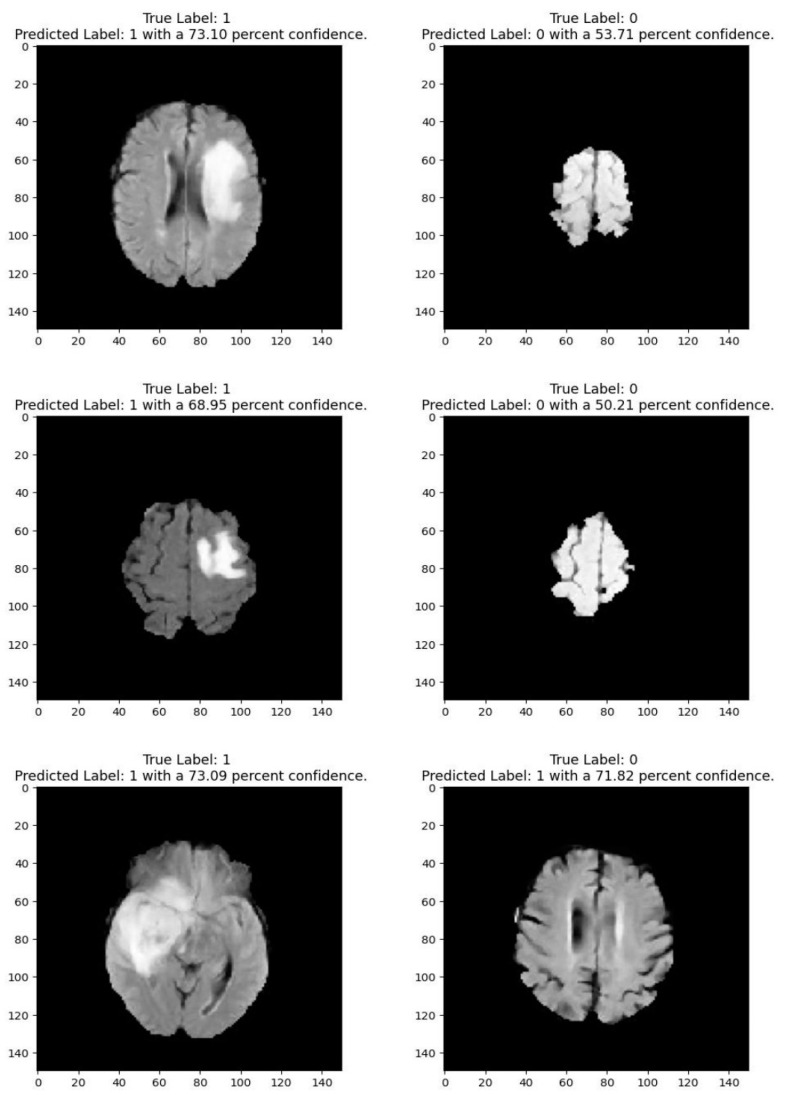
Prediction for tumor (1/0) using fine-tuned transfer learning: VGG19.

**Figure 20:**
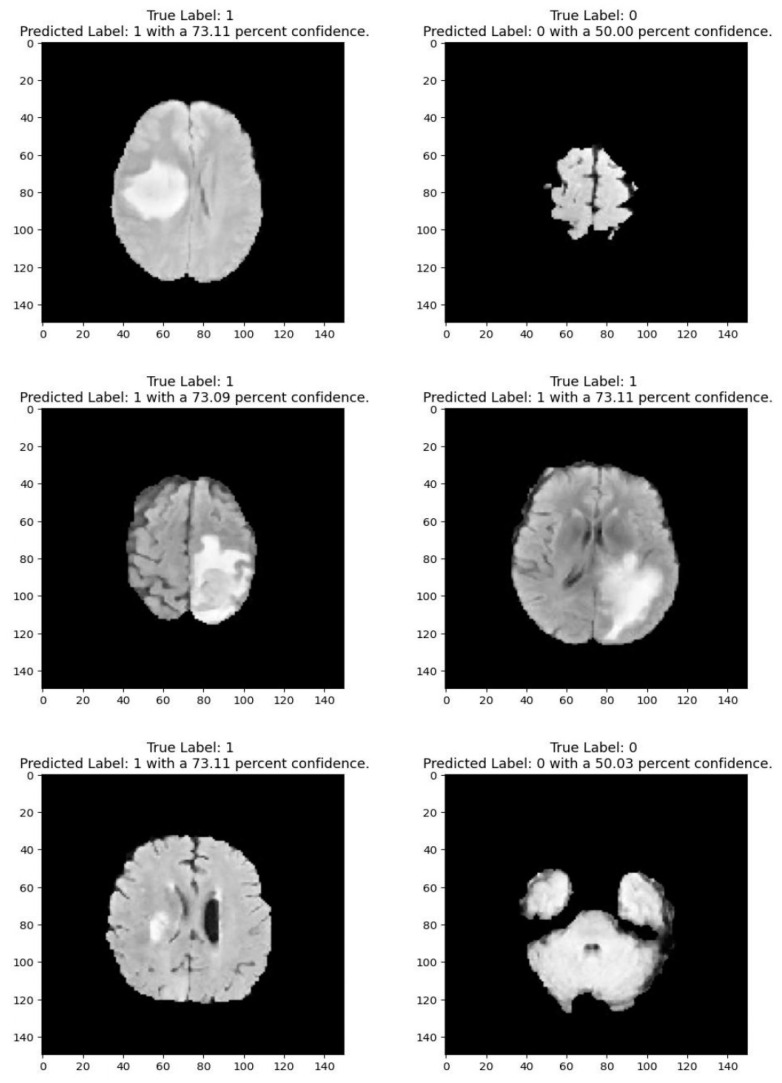
Prediction for tumor (1/0) using fine-tuned transfer learning:s InceptionResNetV2.

Medical imaging encompasses a diverse array of variations, underscoring the significance of image detection in their interpretation. Our focus was on utilizing MRI scans for the detection of brain tumors. MRI serves as a common modality for both detecting and categorizing brain tumors. In our study, we opted to employ fine-tuned transfer learning models to aid in the identification of brain tumors due to their ability to make precise predictions regarding tumor cells. Figure 9, Figure 10, Figure 11 and Figure 12 display graphical representations of the base model combined with transfer learning operations, and Table 4 presents the corresponding findings. Figure 13, Figure 14, Figure 15 and Figure 16 illustrate the epoch-by-epoch progress of the proposed model, with Table 5 summarizing the associated results. We present a comparison in Table 6 of this study, contrasting the existing research with our proposed approach. Figure 17, Figure 18, Figure 19 and Figure 20 represent the prediction of the model in terms of tumor or non-tumor. The model, named Fine-Tuned Transfer Learning Xception, achieved the highest accuracy of 0.9611, as indicated in the table.

This research signifies a significant advancement in the field of medical image analysis, particularly in the realm of brain tumor detection and prediction using magnetic resonance imaging (MRI) data. The contribution of this research lies in its meticulous exploration and evaluation of several cutting-edge deep learning architectures—InceptionResNetV2, VGG19, Xception, and MobileNetV2—within the context of a fine-tuned transfer learning framework.

Firstly, the utilization of deep learning architectures like InceptionResNetV2, VGG19, Xception, and MobileNetV2 represents a sophisticated approach to feature extraction and representation learning from MRI images. These architectures are highly regarded for their ability to extract complicated patterns and characteristics from large amounts of data, which makes them ideal for jobs involving the interpretation of medical images. To further improve the models’ generalization and resilience, this study uses augmentation strategies. In order to artificially improve the variety in the training dataset, augmentation entails performing different transformations, such as rotation, scaling, flipping, or adding noise, to the input data.

Secondly, the integration of these architectures within a fine-tuned transfer learning framework is crucial. Transfer learning allows leveraging knowledge gained from pre-trained models on large datasets (typically non-medical) and adapting it to the specific task at hand—brain tumor detection and prediction in this case. Fine-tuning involves retraining the pre-trained models on a smaller dataset of MRI images, thereby customizing the learned features to better suit the characteristics of medical images.

The comparative analysis of the brain tumor detection models highlights the varying performance of different architectures based on methodology and dataset. Traditional deep learning models such as a CNN (92.67% on BraTS 2018) and a DNN (93.10% on BraTS 2014) have shown strong results, while pre-trained architectures like VGG-19 (94.82% on CE-MRI) and Pre-trained CNN (94.58% on CE-MRI) have further improved accuracy by leveraging transfer learning. More advanced hybrid approaches, such as NS-CNN (95.62%) and XG-Ada-RF (95.90%), utilize ensemble techniques to enhance classification performance. The proposed fine-tuned transfer learning models demonstrate competitive and superior performances compared to prior studies. Among them, Xception achieves the highest accuracy of 96.11%, surpassing even ensemble-based techniques. This can be attributed to its depthwise separable convolutions, which optimize feature extraction while reducing computational complexity, making it highly effective for MRI-based classification. Additionally, MobileNetV2 (94.48%), VGG19 (92.46%), and InceptionResNetV2 (92.26%) further validate the effectiveness of fine-tuned transfer learning.

Despite these promising results, model robustness remains a key concern. The fine-tuned Xception model achieves a 96.11% accuracy, outperforming several state-of-the-art architectures, indicating its superior feature extraction capability. Robustness is further ensured through transfer learning, where pre-trained weights from large-scale datasets enhance the model’s ability to generalize across different types of brain tumors. To prevent overfitting and improve generalization, the model incorporates data augmentation techniques such as rotation, flipping, and contrast adjustments, ensuring it learns meaningful features rather than memorizing specific patterns. Additionally, regularization techniques like dropout and batch normalization are applied to stabilize training and mitigate variance issues.

By integrating these deep learning architectures within the transfer learning framework, this research aims to achieve several key objectives, as follows:

- Enhanced Detection Accuracy: By leveraging the learned representations from pre-trained models and fine-tuning them on MRI images, the aim is to improve the accuracy of detecting brain tumors. The diverse architectures offer different perspectives on the data, potentially capturing a broader range of tumor characteristics and improving the overall detection performance.
- Improved Prediction Performance: Beyond simple detection, the research also focuses on predicting aspects of the tumors, such as their growth patterns, malignancy, or response to treatment. By harnessing the capabilities of these deep learning architectures, the goal is to develop more accurate predictive models that aid in clinical decision making and treatment planning.
- Robustness and Generalization: The evaluation of multiple architectures allows for a comprehensive assessment of their performance across different datasets and scenarios. This helps in identifying which architectures are most effective for brain tumor analysis and understanding their generalization capabilities across various imaging modalities and patient populations.

The clinical applicability of the proposed model lies in its high accuracy, generalizability, and efficiency in brain tumor detection, making it a valuable tool for medical diagnosis. With the Xception-based model achieving a 96.11% accuracy, it demonstrates a strong potential for assisting radiologists in identifying brain tumors from MRI scans with high precision and minimal error rates. This can significantly reduce diagnostic time, aiding in early detection and treatment planning.

The computational cost and inference speed are crucial factors for real-world deployment, as deep learning models require significant processing power, which may limit their usability in low-resource clinical settings. While our proposed models achieve high accuracy, their feasibility in real-time applications depends on optimizing computational efficiency. Future research can focus on model pruning, quantization, and knowledge distillation to reduce model size and inference time without compromising performance. Additionally, leveraging hardware accelerators, like GPUs, TPUs, or edge computing devices, can enhance the processing speed, making rapid diagnosis more practical. To further improve clinical applicability, integrating optimized models into cloud-based diagnostic platforms or AI-assisted MRI analysis systems can facilitate real-time tumor detection. Conducting real-world trials in hospitals and benchmarking against lightweight architectures like MobileNet and EfficientNet will ensure scalability and efficiency for broader medical adoption.

## 6. Conclusions

This research marks a significant advancement in medical image analysis, particularly in brain tumor detection and prediction using MRI data. A key contribution is the thorough evaluation of state-of-the-art deep learning architectures—InceptionResNetV2, VGG19, Xception, and MobileNetV2—within a transfer learning framework. These architectures enable the extraction of intricate patterns from complex MRI images, while augmentation techniques enhance model robustness and generalization. Fine-tuning allows the models to adapt pre-trained knowledge to brain tumor detection, refining learned features for improved performance. Among the evaluated models, Xception demonstrated the highest accuracy and lowest training loss, indicating strong learning capabilities, while VGG19, InceptionResNetV2, and MobileNetV2 showed competitive validation performance. In terms of practical implications, the proposed models can aid radiologists in automated tumor detection, reducing diagnostic time and enhancing decision making in clinical settings. To facilitate hospital adoption, future work will focus on optimizing computational efficiency for real-time deployment, integrating the model into cloud-based diagnostic platforms, and ensuring compatibility with hospital imaging systems. Additionally, further improvements will include refining data augmentation strategies, enhancing generalization across diverse patient demographics, and validating model performance on larger, multi-center datasets. These advancements will help bridge the gap between research and real-world medical applications, ultimately improving early diagnosis and patient outcomes.

## Figures and Tables

**Table 1:** Numbers of images belonging to the two classes.

Tumor Class	Denoted Label	Number of MRI Images
Tumorous	1	1683
Non-Tumorous	0	2079

**Table 2:** Distribution of the dataset.

Set	Denoted Label	Number of MRI Images
Training	1	1822
	0	1187
Testing	1	138
	0	239
Validation	1	119
	0	257

**Table 3:** Hyperparameters of all exploit transfer learning models.

Metric	Metric Value
Batch size	32
Optimizer	Adam
Epochs	30
Learning rate	0.0001 For TL and 0.0001/10 for fine tunning
Criterion	Binary cross-entropy

**Table 4:** Performance matrices for base model + transfer learning.

Model	Training		Validation	
	Accuracy	Loss	Accuracy	Loss
InceptionResNetV2 + Added Layer	0.9226	0.1919	0.9016	0.2872
VGG19 + Added Layer	0.9146	0.2181	0.8298	0.4668
Xception + Added Layer	0.9435	0.1450	0.8910	0.3190
MobileNetV2 + Added Layer	0.9322	0.1560	0.9043	0.2980

**Table 5:** Performance matrices for fine-tuned transfer learning model.

Model	Training		Validation	
	Accuracy	Loss	Accuracy	Loss
Fine-Tuned Transfer Learning InceptionResNetV2	0.9226	0.1919	0.9016	0.2872
Fine-Tuned Transfer Learning VGG19	0.9246	0.1852	0.8511	0.3495
Fine-Tuned Transfer Learning Xception	0.9611	0.0925	0.9096	0.2771
Fine-Tuned Transfer Learning MobileNetV2	0.9448	0.1467	0.9069	0.2751

**Table 6:** Comparison with other state-of-art model.

Source	Methodology	Dataset	Accuracy
[46]	Faster R-CNN with Transfer Learning	-	0.9300
[47]	Dense Fused Maxout Network	BraTS and Figshare	0.9200
[48]	CNN	BraTS 2018	0.9267
[30]	VGG16, InceptionV3, and ResNet50	-	0.9158
[49]	VGG-19	CE-MRI 3064 images from 233 patients.	0.9482
[50]	Custom CNN architecture, BAT Algorithm	BraTS2015	0.9200
[51]	U-Net architecture GANs	TCGA-GBM and TCGA-LGG	0.8882
[52]	Stack autoencoder in DL	BraTS 2015	0.9500
[53]	DNN	BraTS2014	0.9310
[54]	Pre-trained CNN	CE-MRI	0.9458
[55]	Active DNN	BraTS2018	0.9250
[56]	NS-CNN	TCGA-GBM	0.9562
[57]	BRAIN-TUMOR-net	-	0.9367
[58]	XG-Ada-RF (Ensemble of Extreme Gradient Boosting, Ada-Boost, and Random Forest)	Figshare	0.9590
[59]	VGG16 + RESNET	Kaggle, Figshare, SARTAJ, Br35H	0.9200
Proposed	Fine-Tuned Transfer Learning InceptionResNetV2	Kaggle	0.9226
Proposed	Fine-Tuned Transfer Learning VGG19	Kaggle	0.9246
Proposed	Fine-Tuned Transfer Learning Xception	Kaggle	0.9611
Proposed	Fine-Tuned Transfer Learning MobileNetV2	Kaggle	0.9448

## Data Availability

Data will be made available in “Brain tumor [WWW Document], 2020. Kaggle. URL https://www.kaggle.com/datasets/jakeshbohaju/brain-tumor”.
